# Mordellidae (Coleoptera) Research: A Review Based on the *Zoological Record* from 1864 through 2013

**DOI:** 10.3390/insects9030113

**Published:** 2018-09-03

**Authors:** Yang Liu, Terry L. Erwin, Xingke Yang

**Affiliations:** 1 Key Laboratory of Resource Biology and Biotechnology in Western China (Northwest University), Ministry of Education, College of Life Science, Northwest University, Taibai North Road 229, Xi’an 710069, China; 2 Key Laboratory of Zoological Systematics and Evolution, Institute of Zoology, Chinese Academy of Sciences, Beijing 100101, China; 3 Department of Entomology, National Museum of Natural History, Smithsonian, Washington, DC 20013, USA; erwint@si.edu

**Keywords:** Mordellidae, academic literature review, classification framework, Zoological Record, list of generic taxa with citation

## Abstract

Mordellidae (tumbling flower beetles) is a globally distributed family of Coleoptera; it is among the most species-rich families (containing 115 genera and 2308 species described). It is important because of its agroforestry significance and its ecosystem-sustaining attributes. However, the past and current status of Mordellidae research remains unclear. A comprehensive literature review of Mordellidae articles published over the period of 1864–2013 based on the *Zoological Record* was conducted for the first time. A total of 863 articles were used for analysis after a strict literature search using screening protocols. These articles were then assigned to four categories based on the year of publication, topics/themes, primary authors, and frequently utilized journals for publication of Mordellidae-related articles. The results reveal that: (1) there are three prosperous research periods (1876–1898, 1922–1957, and 1977–2012) for Mordellidae during 1864–2013 that are associated with the active period of three generations of the main taxonomists. However, it is unfortunate that it also demonstrates there is a lack of upcoming researchers to continue the work after the retirement of the current generation, thus action should be taken immediately to promote research on Mordellidae; (2) on average, each primary author published 3.1 papers, but ~35% of the Mordellidae articles were published by less than 3% of the primary authors; (3) researchers tended to mostly publish their articles in local journals of their home countries; (4) more than 90% of the articles pertain to traditional taxonomy, with those of early times generally containing only simple descriptions of the species and the holotypes chosen are sparsely deposited with the researchers or amateurs around the world, thus making them difficult to be checked; (5) nearly half of the studies described Mordellidae species from Palaearctic realm, about one-third of the studies described species in other areas rather than in the fauna in which the authors lived, and about two-fifths of the studies described species from countries outside of the authors’ country of origin. Therefore, the in-depth systematic study of worldwide Mordellidae is required to reconstruct Mordellidae phylogeny and a revision of its classification system with modern methods of comparative morphology, molecular biology, zoogeography, and cladistics. In order to better understand the life stages and biology of Mordellidae insects, more work on Mordellidae ecology should be undertaken to develop strategies for pest control. We hope that this review will provide information to the novice and expert alike in Mordellidae research pertaining to its past and current status, possible future research areas, and attract more attention from the scientific world and renew an interest in Mordellidae research.

## 1. Introduction

The Mordellidae (also called tumbling flower beetles) is a family of beetles with a great number of species. More than 2308 Mordellidae species belonging to 115 genera have been recorded so far and more species are appearing in the literature often, including 45 new species in the past 10 years. Mordellidae are found worldwide in a variety of ecosystems, from tropical rainforests to arctic tundra, and from sea level to elevations of above 4000 m (>13,123 ft). Adult Mordellidae are found on dead or partly dead trees and a great many of them are pollinators [1,2]. While generally their larvae feed on the sap of vascular plants, some larvae are also found in dead trees, or still others bore in the pith of plants, hard mushrooms, and some are even predaceous on other larvae (e.g., lepidopterous and dipterous larvae) [1,3]; some of them are therefore important agricultural and forestry pests.

Linneaus is considered to be the Mordellidae pioneer who established *Mordella*, the first genus of Mordellidae, in 1758 [2]. However, it was not until 1802 that Mordellidae was actually established by Latreille [4], which marks the start of the traditional taxonomy of Mordellidae. It is should be noted that for a long period of time (until 1855), Ripiphoridae were considered a part of Mordellidae. However, current molecular studies provide weak support for this monophyly [5,6], which is in contradiction with the morphology and biology of the groups [5,7]. A considerable amount of work has been done by investigators around the world since then. It is of paramount importance to collate and synthesize these previous studies in order to provide a general picture of historical Mordellidae research that will serve future research [8,9]. However, to our knowledge, no one has collated and synthesized these works. The objective of this study is therefore to understand the state of Mordellidae research by examining the published literature. Based on the *Zoological Record*, publications pertaining to Mordellidae from 1864 through 2013 were screened and analyzed to provide insights for Mordellidae practitioners and researchers alike on the major turning points of historical trends in and the implications for the future directions of Mordellidae research, as well as to compile a systematic reference to the Mordellidae literature.

Although many genera/species (e.g., *Conalia* (Mulsant & Rey, 1858), *Ctenidia* (Laporte de Castelnau in Brullé, 1840), *Curtimorda* (Naezen, 1794), *Hoshihananomia* (Sulzer, 1776), *Mordella* (Linnaeus, 1758; Eichwald, 1830), *Mordellistena* (Linnaeus, 1758; Panzer, 1796; Fabricius, 1798; Gyllenhal, 1810, 1819, 1827), *Mordellocbroa* (Fabricius, 1775), *Tolida* (Mulsant, 1856), *Tomoxia* (Gyllenhal, 1827; Rossi, 1794), and *Variimorda* (Schrank, 1781; Comolli, 1837)) were described between 1758 and 1864 [4], pioneer research within this period is not included due to the following reasons: (1) there is a lack of literature and limited access to holotypes of the previously described species [2]; (2) insufficient descriptions (e.g., undescribed or rarely mentioned sexual divergences) given by previous researchers make it difficult to identify and classify this family [1]; and (3) many specimens were incorrectly identified and the system of taxonomic arrangement has been changed by later researchers [1,4].

## 2. Methods for the Literature Search

The scope of this investigation was limited to the time frame 1864–2013 because publications in various journals of this period were recorded in the *Zoological Record*, a ready source of taxonomic literature with coverage back to 1864 (published annually by Thomson Reuters). *Zoological Record* can be used to determine the first appearance of an animal in the published literature, track the changes in classification, and monitor the developments in ecology, conservation, and wildlife management/preservation. The literature recorded by the *Zoological Record* also (1) mirrors the developments and evolution in techniques and methods for Mordellidae research and (2) reflects the renewal of researchers’ interest in such research after the adoption of new techniques, as will be discussed in next section.

The literature search was based on the descriptor “Mordellidae” in the title, key words, and/or abstract in the *Zoological Record*. The full text or abstract of each article was reviewed to eliminate articles that were not related or when only a minor section was about Mordellidae.

## 3. Results and Discussion of the Analysis

Our literature search and screening, performed according to the established procedures and criteria, returned 863 published papers in 300 different journals during the period 1864–2013. We categorized this literature in a few different ways, such as year of publication, journals, topics, authors and their affiliated countries, and geographic origin of described species, and these categories are analyzed and discussed accordingly below. This particular analysis should provide guidelines for the pursuit of future research on Mordellidae and its applications by explaining the chronological growth of Mordellidae over the years, the challenging areas of Mordellidae research, and the major issues surrounding taxonomy, ecology, pest control, and phylogeny.

### 3.1. Distribution of Articles by Year of Publication

**Figure 1:**
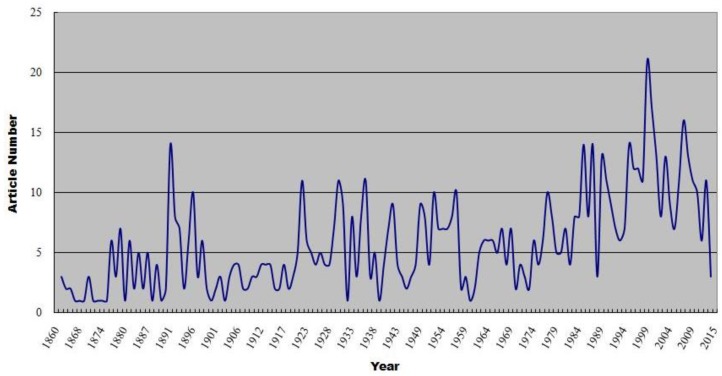
Annual number of articles on Mordellidae over the period from 1864 through 2013.

The article distribution pattern by year of publication (from 1864 through 2013) is illustrated in Figure 1. It shows a growing trend in the research on Mordellidae over this period and is marked by three high-volume periods: (1) 1876–1898; (2) 1922–1957; and (3) 1977–2012. Fluctuations of publications during these three periods are observed in each period and the greatest number of articles is found in 1999 (21). These three high-volume periods are associated with the active period of some of the main taxonomists, or three generations of Mordellidae researchers. For example, the active period of Pic, M. (1899–1954), Champion, G.C. (1890–1927), and Fairmaire, L. (1891–1906), who were the main contributors for the first high-volume period, as shown in Figure 1. Ermisch, K. (1940–1972) and Franciscolo, M. (1942–2001) contributed to the second high-volume period. Horak, J. (1978–2012), Takakuwa, M. (1976–2010), and Odnosum, V.K. (1983–2003) made the third high-volume period.

The number of articles grew more quickly than ever since the early 1980s through 2013, after which the number of Mordellidae articles fell precipitously, which could be attributed to the retirement of nearly all of the most prolific tumbling flower beetle taxonomists, mainly in European countries. Unfortunately, these eminent researchers have not been replaced by a new generation of Mordellidae experts [2]. The situation of Mordellidae research is much different from other fields such as leafhoppers, which has had a large group of young taxonomists in recent years [8].

### 3.2. Distribution of Articles by Journal

The number of journals under the category of insect sciences has been increasing considerably, from 87 journals in 2000 to 127 journals in 2010 and 143 journals in 2017 (accessed on 11 August 2018). However, only a portion of the journals have been utilized by Mordellidae researchers. Table 1 illustrates the distribution of Mordellidae articles by journal. It should be noted that there are 43 journals that published over 10 Mordellidae papers, mostly on topics of taxonomy. *Vestnik Zoologii* published 54 Mordellidae related articles in total, followed by *Bulletin de la Societe Entomologique de France* with 52 articles, and then *Elytra* with 50 articles. It is interesting to notice that the authors of these articles are mainly from countries where the journals were established, as will be discussed below. In these more utilized journals, a great number of previous works were dedicated to classification. Considering the changes in the recent trend in the publication of Mordellidae articles, a brief introduction to some of the most utilized journals is given below for the reader’s reference.

*Vestnik Zoologii* (Zoological Herald) is a bimonthly journal founded in 1967 in Ukraine; it publishes original papers in all fields of zoology. We found that all of the 54 Mordellidae articles published in this journal are from V.K. Odnosum (1983–2009), who is also a Ukrainian.

*Bulletin de la Societe Entomologique de France* was officially founded in 1832 with the sponsorship of the *Entomological Society of France*, which is the oldest entomological society in the world. There are 52 Mordellidae articles published over the period from 1868 to 2001. The main taxonomists are A. Chobaut (first and last year of publication on this journal: 1894–1924, six articles) and M. Pic (1899–1942, eight articles).

*Elytra* is the third top journal that has published Mordellidae articles. This journal was established in 1973 and is sponsored by the Coleopterological Society of Japan; it has published 50 articles about Mordellidae until now. M. Takakuwa (1976–2010, 19 articles) and T. Tsuru (2004–2012, 5 articles, including 3 articles that are coauthored with M. Takakuwa) are the main researchers. *Coleopterists’ News* (1968–2010, in Japanese) is the other journal sponsored by the Coleopterological Society of Japan; it has published 11 Mordellidae articles. All the Mordellidae articles published in this journal from 1998 to 2007 are by M. Takakuwa.

*The Entomologist’s Monthly Magazine* is a British entomological journal that started publication in 1864. The journal publishes original papers and notes on all orders of insects and terrestrial arthropods from around the world, specializing in groups other than Lepidoptera. There are 46 articles published in this journal, with the main contributor being G.C. Champion (1891–1927, 11 articles) and K.G. Blair (1922–1934, 5 articles).

### 3.3. Distribution of Articles by Topic

Articles are divided into five categories based on the topics/theme, including traditional taxonomy, molecular, bioinformatics, ecology, and pest control. Each category is discussed separately and some good examples are given below:

(1) **Traditional taxonomy** classifies Mordellidae according to their morphological attributes. More than 90% of these articles were found to be traditional morphology based, with simple descriptions of new species only. The preponderance of taxonomists at the beginning of 18th century was European; after that, researchers from Asia and America started working on them. Some milestone research works worth attention over the traditional taxonomy period are: (1) Khalaf [10] and Odnosum [11], who discussed wing venation of Mordellidae and found phylogenetic relationships in Mordellidae by the structure of hindwings; (2) Lu et al. [12] compared male genitalia of 12 different genera and found phylogenetic relationships in North American Mordellidae; (3) Odnosum [13,14,15,16,17], who discussed several morphological characteristics of Mordellinae, including antennae, mouthparts, sternum, structure of wings, male genitalia, etc. Instead of describing only the maxillary palp of males, as did most researchers, Odnosum [18,19] described other morphological characteristics of both male and female Mordellidae. There are also important studies of M. Franciscolo and many others which are not mentioned above.

(2) **Ecologists** investigate the life history of Mordellidae species and their biology (e.g., plants, water, temperature, natural hazards, and other insects). Life history is critical to the study of disease transmission. Hayashi [20] showed that larvae of some genera of Mordellidae live in dead trees in Japan. Mamayev and Odnosum [21] presented new data on morphology and systematics of five species of Mordellidae larvae of Far Eastern USSR fauna. Ford and Jackman [22] connected 11 species of Mordellidae and 22 species of hosts and reported new larval host plant associations in North America. Zemoglyadchuk [23] reported the morphological attributes of larvae of three species of *Mordellistena parvula* groups and stressed the importance of studying larval hosts to identify species of Mordellidae. Identification of larval hosts may also facilitate understanding the life cycle of Mordellidae species and the development of pest control strategies.

(3) **Pest control**. Mordellidae are of significant agroforestry importance, e.g., many species are pollinators, while some species are pests that lead to great economic losses. Therefore, better understanding of the classification and ecology of Mordellidae can facilitate the development of strategies for pest control. Unfortunately, few articles found in the *Zoological Record* reported the threats and prevention of Mordellidae. The few available reports are about their risk to sunflowers. Fan [24] stated the reason for the investigation and recording of new species of Mordellidae was that Mordellidae are harmful to the local sunflower industry in China. Qureshi et al. [25] reported that plant populations and weeds influence stalk insects (Mordellidae), soil moisture, and yield in rain-fed sunflowers.

(4) **Fossil studies**. Fossil Mordellidae are rarely found and reported [26], but they can be valuable to understanding the evolutionary history of Mordellidae. The earliest Mordellidae fossils found in the Karatau Range in Kazakhstan were considered to be from the Late Jurassic; it was described as a new genus and species, *Praemordella martynovi* Scegoleva-Barovskaja [27]. Based on this, Scegoleva-Barovskaja [27] defined a new subfamily Praemordellinae under the family Mordellidae. Medvedev [28] established a new genus (*Scraptiomima*) based on the recovered fossils in the Transbaykalia region of USSR. In 1993, Wang [29] established the monotypic family Liaoximordellidae based on a fossil from Liaoning, China. This family is now regarded as a fossil genus, Liaoximordella of the Mordellidae. In addition, Jell and Duncan [30] also reported Mordellidae fossils in Victoria, Australia. Huang and Yang [26] established a new genus (*Cretansapis*) based on fossils recovered at Fangshan, Beijing, China and discussed the evolution and taxonomic position of Mordellidae. Cockerell [31] reported a few fossil insects deposited at the United States National Museum, in which there was a new species of Mordellidae, *Mordella priscula* Cockerell 1925. Other researchers, such as Statz [32], Nel [33], Kubisz [34], Liu et al. [35,36], Peris and Ruzzier [37], and Odnosum [38], also reported new fossil/amber Mordellidae species, or established a new genus based on the described species.

### 3.4. Distribution of Articles by Author

**Figure 2:**
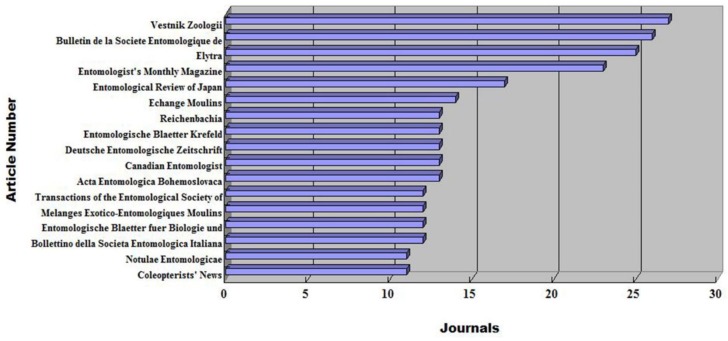
The distribution of articles by journal.

There are 268 primary authors for the 863 articles, which is ~3.2 papers on average for each author over the last 150 years (1864–2013). Over half of the 863 papers were published by researchers who published 10 or more Mordellidae articles. As Figure 2 shows, the top contributors are: M. Pic (48 articles, the period of his related research from 1899 to 1954), K. Ermisch (58 articles, the period from 1940 to 1972), M. Franciscolo (57 articles, 1942–2001), J. Horak (41 articles, 1978–2012), M. Takakuwa (38 articles, 1976–2010), V.K. Odnosum (33 articles, 1983–2009), and G.C. Champion (23 articles, 1890–1927). These seven researchers (3% of the 268 primary authors) published over one-third of the total articles (298), which may indicate that Mordellidae may be the expertise or focus of a very small portion of these authors. A summary of articles for the significant contributors is shown in Table 2.

We also investigated the country origins of the primary authors (Table 2). The results showed that Mordellidae research is heavily internationalized, with the majority of authors from European and Asian countries.

### 3.5. Distribution of Geographic Origin of Described Species

We investigated the distribution of the geographic origin of described species by animal fauna (i.e., Palearctic realm, Nearctic realm, Neotropical realm, Ethiopian realm, Oriental realm, and Australian realm). There are 401 Mordellidae studies related to species in the Palearctic realm, 114 in the Oriental realm, 90 in the Ethiopian realm, 88 in the Nearctic realm, 59 in the Australian realm, and 49 in the Neotropical realm. Twenty-two studies described species from two realms, two publications described species from more than two realms, and the rest focused on species from a single realm. It is interesting to note that more than 250 studies described species from other animal fauna rather than the fauna from the region to which the authors belonged. This might be evidence of increased international collaboration or lack of described species from authors’ own realms. By comparing the country of origin for the described species and authors, we found that more than 350 studies described species from countries outside of the authors’ country of origin.

## 4. Conclusions and Future Studies

Mordellidae is among the most important families of Coleoptera, with 115 genera and 2308 species distributed worldwide. These taxa play critical roles in the function and sustainability of the ecosystem. The larvae of some species are important pests for agricultural and forestry industries, while at the same time the adults are beneficial pollinators. In order to obtain a general picture of global mordellid research, we undertook a comprehensive review of articles on the family covering the period from 1864 through 2013 based on the *Zoological Record*. To the best of our knowledge, this is the first academic literature review on Mordellidae research depicting the past and current status of the family [2].

A total of 863 articles were selected for analysis following strict literature search and screening protocols. These articles were then divided into four categories based on the year of publication, journals, topics/themes, and primary authors. Our results demonstrate the following:

(1) In these 863 papers, traditional taxonomy is the dominant topic, accounting for more than 90% of the total articles. In addition, literature of earlier times generally contains only simple descriptions of the species, with these being less likely to be used for phylogenetic classification. The holotypes used were non-discretely deposited around the world, making future research more difficult.

(2) There are 268 primary authors for the 863 articles, with ~3.1 papers on average for each author over the last 150 years (1864–2013). It is interesting to note that ~36% of the Mordellidae articles were published by only 3% of the primary authors, which may indicate that only a few researchers have focused on this family and the rest of them did not attempt to conduct in-depth and systematic studies of Mordellidae. The underlying reason for this remains unknown.

(3) Mordellidae research had three high-volume periods, and these were associated with the active periods of some of the main taxonomists, i.e., three generations of Mordellidae researchers.

(4) We found the distribution of articles by journal to be very interesting. Early publications were made by authors mainly from countries where the journals were established. Later, there has been an increasing trend toward publishing in international journals.

(5) The Palearctic realm is the most frequently studied region for Mordellidae, accounting for about half of the studies, while about one-third of the studies described species from other animal fauna rather than the fauna from the region to which the authors belonged. Similarly, we found that 40% of the studies described species from countries outside of the authors’ country of origin.

Our conclusions based on the literature analysis are that many genera and species need to be revised, especially old Mordellidae descriptions. Further, new species need to be described, especially those in less studied regions, such as the Oriental realm, the Ethiopian realm, the Nearctic realm, the Australian realm, and the Neotropical realm. Along with these revisions and new descriptions, there is a need for an in-depth systematic study of worldwide Mordellidae, which is of paramount importance for modern phylogenetic reconstruction and an overhaul of the family’s classification system. Using modern methods of comparative morphology, molecular biology, zoogeography, and cladistics will secure a better understanding of the world’s Mordellidae fauna. A list of the Mordellidae generic taxa can be found in the Appendix A.

Furthermore, the employment of molecular techniques has been proven to significantly improve the accuracy of taxonomy of many other families of Insecta [39]. Thus, phylogenetic studies based on both morphological and molecular data should be the main direction for future studies, especially at the species level, where distinct morphological characteristics are difficult to identify. Additionally, attention should also go to the study of Mordellidae’s way of life, so as to better understand the life stages of tumbling flower beetles and their relationships to environments; this can also serve as the foundation for developing strategies for pest control.

However, as our literature review amply demonstrates, the retirement of the third generation of eminent Mordellidae researchers has unfortunately left a research vacuum, as nearly no one is taking over their research tasks. In addition, there are no large-scale funds or training programs for taxonomists in Mordellidae and the lack of taxonomists and funds have also been reported in other research in Insecta and biodiversity [40,41,42,43,44]. We therefore believe actions should be undertaken immediately to promote the research of Mordellidae and attract more attention from the scientific world. We hope that this lengthy literature review and analysis of the field of Mordellidae research will be the beginning of a resurgence.

## Figures and Tables

**Table 1:** The distribution of articles by journals.

Journals	Article Number
Vestnik Zoologii	54
Bulletin de la Societe Entomologique de France	52
Elytra	50
Entomologist’s Monthly Magazine	46
Entomological Review of Japan	34
L’Echange, Revue Linnéenne	28
Acta Entomologica Bohemoslovaca	26
Canadian Entomologist	26
Deutsche Entomologische Zeitschrift	26
Entomologische Blaetter Krefeld	26
Reichenbachia	26
Bollettino della Societa Entomologica Italiana	24
Entomologische Blaetter fuer Biologie und Systematik der Kaefer	24
Melanges Exotico–Entomologiques Moulins	24
Transactions of the Entomological Society of London	24
Notulae Entomologicae	22
Coleopterists’ News	20
Annales de la Societe Entomologique de France	18
Coleopterists Bulletin	18
Entomologische Berichten	18
Entomologist’s Record and Journal of Variation	18
Proceedings of the Linnean Society of New South Wales	18
Annales de la Societe Entomologique de Belgique	15
Acta Societatis Zoologicae Bohemicae	14
Berliner Entomologische Zeitschrift	14
Memorie della Societa Entomologica Italiana	14
Annali del Museo Civico di Storia Naturale “Giacomo Doria”	12
Annals & Magazine of Natural History	12
Bulletin of the Osaka Museum of Natural History	12
Coleopterist	12
Environmental Entomology	12
Insecta Matsumurana Sapporo	12
Kaefer Europas	12
Pan–Pacific Entomologist	12
Proceedings of the Royal Entomological Society of London	12
Special Bulletin of the Japanese Society of Coleopterology	12
Studies and Reports of District Museum Prague–East Taxonomical Series	12
Transactions of the Royal Society of South Australia	12
Annals of Natural History	10
Arkiv for Zoologi Stockholm	10
Bulletin of the Brooklyn Entomological Society	10
Ecological Entomology	10
Revue Francaise d’Entomologie	10
Total	863

**Table 2:** The distribution of articles by primary authors, years, and countries.

Name	Period	Article Number	Country
Pic, M.	1899–1954	64	France
Ermisch, K.	1940–1972	58	Germany
Franciscolo, M.	1942–2001	57	Italy
Horak, J.	1978–2012	41	Czech
Takakuwa, M.	1976–2010	38	Japan
Odnosum, V.K.	1983–2009	33	Ukraine
Champin, G.C.	1890–1927	23	Britain
Fairmaire, L.	1891–1906	18	France
Batten, R.	1976–1990	15	The Netherlands
Ray, E.	1930–1947	15	USA
Blair, K.G.	1915–1942	13	Britain
Nomura, S.	1957–1967	11	Japan
Lea, A.M.	1895–1931	11	Australia
Nakane, T.	1949–1960	10	Japan
Kolbe, H.	1910–1934	10	German
Chobaut, A.	1892–1924	10	France
Kangas, E.	1976–1988	9	Finland
Schilsky, J.	1895–1910	9	Germany
Tsuru, T.	2002–2012	8	Japan
Shiyake, S.	1994–2001	8	Japan
Allen, A.A.	1975–2007	8	Britain
Chujo, M.	1935–1964	8	Japan
Roubal, J.	1921–1935	7	German
Kiyoyama, Y.	1875–2010	7	Japan
Lu, W.	1997–2009	6	America
Kubisz, D.	2000–2010	5	Poland
Liljeblad, E.	1917–1945	5	USA
Wickham, H.F.	1909–1914	5	USA
Xambeu, V.	1893–1908	5	France
Blackburn, T.	1891–1893	5	Australia

## Appendix A

**Table A1:** List of Mordellidae Generic Taxa.

Family	Subfamily	Tribe	Genus	Sugenus	Citation
Mordellidae					Latreille, 1802 (Seidlitz, 1875) Franciscolo, 1942 Crowson, 1953 Franciscolo, 1956: 2 Franciscolo, 1962: 98 Serrahima Ignasi, 2007: 560
	Ctenidiinae				Franciscolo, 1951a: 56
			*Ctenidia*		Laporte de Castelnau in Brullé, 1840 Franciscolo, 1951a: 56
	Mordellinae				Latreille, 1802 (Fowler, 1912) Chujo, 1935: 75 Chujo, 1935: 78 Chujo, 1935: 80, 82 Chujo, 1935: 84 Franciscolo, 1942: 6, 8 Franciscolo, 1952 Franciscolo, 1953 Franciscolo, 1955: 1052 Franciscolo, 1956: 2 Franciscolo, 1959: 23 Franciscolo, 1962: 98 Serrahima, 2007: 560
		Conaliini			Ermisch, 1956 Lu, 1997: 756
			*Conalia*		Mulsant & Rey, 1858: 313 Champion, 1896: 49 Champion, 1898: 98 Ray, 1941: 285 Liljeblad, 1945: 16 [Hungary; Czech: Moravia; North America; Europe] Ermisch, 1950: 73 Ermisch, 1956: 304 Downie, 1996: 1155 [North-Eastern America] Lu, 1997: 756
			*Conaliamorpha*		Ermisch, 1968c: 225
			*Glipodes*		LeConte, 1862: 47–48 Emery, 1876: 79 Smith, 1882: 74, 84 [America] Champion, 1891: 305 Blatehley, 1910: 1309, 1315 Ray, 1941: 285 [Mexico] Liljeblad, 1945: 14 [North America] Ermisch, 1950:72 Downie & Arnett, 1996: 1155 [North-eastern America]
			*Isotrilophus*		Liljeblad, 1945: 18 [North America]
			*Jakliella*		Horak, 2010: 558 [Indonesia: West Papua]
			*Javamorda*		Ermisch, 1968: 33–34
			*Jenisia*		Horak, 2008: 78–79 [Madagascar]
			*Larisia*		Emery, 1876: 13 Liljeblad, 1945: 220
			*Lubosiella*		Horak, 2007: 60 [Vietnam]
			*Lycidomorda*		Horak, 2007: 66 [Philippines]
			*Mehlia*		Horak, 2010: 549 [Malaysia: Malayan Peninsula: Perak; Sabah; Indonesia: Kalimantan, Sumatra, Ambon, Seram Island, Tanimbar Archipelago: Yamdena]
			*Mordellista*		French, 1933: 33–35
			*Nassipa*		Emery, 1876: 13 Liljeblad, 1945: 222
			*Nipponanaspis*		Chujo, 1957: 11
				*Konaspis*	Chujo, 1957: 14–16
			*Ophthalmoconalia*		Ermisch, 1968: 268
			*Palmorda*		Ermisch, 1969: 312
			*Paraconalia*		Ermisch, 1968c: 237
			*Paramordellistena*		Ermisch, 1950: 73
			*Pelccotomoides*		Sharp & Muir, 1912: 555
			Pseudoconalia		Ermisch, 1950: 72
			*Pseudomordella*		Ermisch, 1950: 73 [Indonesia: Sumatra]
			*Pseudomordellina*		Nakane, 1957: 49–50
			*Pseudomordellistena*		Ermisch, 1950: 24
				*Mordellina*	Ermisch, 1952: 56
			*Pulchrimorda*		Ermisch, 1968 Horak, 2007: 69 [China; Indonesia; Philippines]
			*Rhipiphorus*		Quedenfeldt, 1886: 128
			*Scaphiostena*		Horak, 1994: 191
			*Scraptia*		Xambeu, 1908: 284
			*Silaria*		Mulsant, 1856: 39, 426 Liljeblad, 1945: 219
			*Sinopalpus*		Horak, 2007: 78 [China]
			*Sphingoccphalus*		Liljeblad, 1945: 206
			*Stenoconalia*		Ermisch, 1967: 130
			*Striganaspella*		Ermisch, 1952: 100
			*Striganaspis*		Ermisch, 1950: 87
			*Xanthoconalia*		Franciscolo, 1942 Franciscolo, 1943: 292 [Congo] Ermisch, 1950: 73
		Mordellini			Siedlitz, 1875 Leconte & Horn, 1883: 408 Ermisch, 1941 Ermisch, 1942 Franciscolo, 1955: 1052 Franciscolo, 1956: 2 Franciscolo, 1959: 23 Franciscolo, 1962: 98 Nomura, 1967: 5 Lu, 1997: 743 Serrahima, 2007: 560 Horak, 2009: 207
			*Adelptes*		Franciscolo, 1965
			*Asiamordella*		Hong, 2002: 126
			*Austromordella*		Ermisch, 1950: 63
			*Binaghia*		Franciscolo, 1943: 1297
			*Boatia*		Franciscolo, 1985: 79 [Ecuador]
			*Caffromorda*		Franciscolo, 1943: 297, 298 Ermisch, 1950: 56 Franciscolo, 1952: 66
			*Calycina*		Blair, 1922: 222 Blair, 1934: 250 Ermisch, 1950: 71
				*Calycellina*	Pic, 1932: 30
			*Cephaloglipa*		Franciscolo, 1952: 350 [New Guinea Island]
			*Congomorda*		Emery, 1876: 8, 10 Ermisch, 1950: 89 Ermisch, 1955: 26 [Congo]
			*Cothurus*		Franciscolo, 1987: 225 Champion, 1891: 259 Champion, 1898: 85 Ermisch, 1950: 48
			*Cretanaspis*		Huang & Yang, 1999: 125
			*Curtimorda*		Méquignon, 1946: 58 Ermisch, 1950: 69 Tokeji, 1954: 3 Ermisch, 1956: 303
			*Dollmania*		Leconte, 1862: 43 Tams, 1930: 174 Ermisch, 1950: 88 Franciscolo, 1961: 15 Franciscolo, 1962: 98
			*Glipa*		LeConte, 1859: 17 Leconte, 1862: 46 Smith, 1882: 79 Champion, 1891: 236 Champion, 1898: 84 Chujo, 1935: 76 Ermisch, 1940: 161–173 Ermisch, 1941: 45 Korschefsky, 1942: 65 Liljeblad, 1945: 20 [America] Ermisch, 1950: 66 Franciscolo 1952: 55, 57 Ermisch, 1965: 197 Takakuwa, 1976: 16 Takakuwa, 1977: 9 Takakuwa, 1977: 5 Takakuwa, 1986: 260 Takakuwa, 1986: 261 Downie & Arnett, 1996: 1155 [North-Eastern America; North-Eastern Mexico] Lu, 1997: 754 Takakuwa, 2000: 1 Takakuwa, 2002: 357
				*Macroglipa*	Nakane, 1960: 17
				*Stenoglipa*	Nomura, 1967: 7
			*Glipidiomorpha*		Franciscolo, 1952: 347 [New Guinea Island] Franciscolo, 1955: 177
			*Higehananomia*		Kono, 1928: 30 Kono, 1935: 123 Ermisch, 1950:70
			*Hoshihananomia*		Kônô, 1935: 124 Mequignon, 1946: 56 Ermisch, 1950: 65 Tokeji, 1954: 3 Franciscolo, 1955: 170 Ermisch, 1956: 302 Lu, 1997: 752
			*Iberomorda*		Méquignon, 1946: 59 [Europe]
			*Ideorhipistena*		Franciscolo, 2000: 395
			*Klapperichimorda*		Ermisch, 1968: 279
			*Larinomorda*		Ermisch, 1968: 263
			*Liaoximordella*		Wang, 1993: 87
			*Machairophora*		Franciscolo, 1943: 34
			*Macrotomoxia*		Píc, 1922: 208 Chujo, 1935: 75 Ermisch, 1950: 51 [Laos; Japan: Tokyo]
			*Mediimorda*		Mequignon, 1946 Ermisch, 1956: 304 Leblanc & Pascal, 2007: 235–250
			*Mirimordella*		Liu, Lu & Ren, 2007
			*Mordella*		Linnaeus, 1758: 420 Tokeji, 1854: 4 Chambers, 1881: 173–175 Broun, 1880: 413 [New Zealand] Linnaeus, 1882: 80 [America] Smith, 1883 [Columbia] Champion, 1891: 273–304 [Central America] Champion, 1891: 122–124 [Europe] Champion, 1895: 266 [Western Australia, Tasmania Island] Champion, 1896: 48 Kolbe, 1897: 254 Champion, 1898: 85 Xambeu, 1908:249 Wickham, 1909: 130 Nicolay, 1914: 30 Champion, 1917: 178 [Seychelles] Kono, 1928: 34 [Japan] Scegoleva-Barovskaja, 1931: 418 Chujo, 1935: 80 Chujo, 1935: 78 Liljeblad, 1945: 30 Mequignon, 1946: 59 [Europe] Normand, 1949: 82 Ray, 1949: 278 [Fiji] Buck, 1954: 16 Ermisch, 1956: 300 Franciscolo, 1956: 2 Horak, 1979: 106 Plaza, 1981: 198 Downie, 1996: 1158 [Northeast America; Northeast Mexico] Lu, 1997: 750 Serrahima & Leblanc, 2007: 560
				*Mediimorda*	Mequignon, 1946: 62
				*Mordella*	Mequignon, 1946: 60
				*Mordellistena*	De Bone, 1865: 278
				*Sulcatimorda*	Mequignon, 1946: 63
				*Variimorda*	Mequignon, 1946: 62
			*Mordellapygium*		Ray, 1930a: 143 Ermisch, 1950: 56
			*Mordellaria*		Ermisch, 1950: 69 Tokeji, 1954: 4 Ermisch, 1956: 303 Franciscolo, 1956: 215 Horak, 1995:81 [South Africa] Lu, 2009: 118 [Africa, America, Asia]
			*Mordellina*		Schilsky, 1908 [5]: 137 Franciscolo, 1962: 107 Ermisch, 1965: 199 Batten, 1990: 150 [Papua New Guinea] Franciscolo, 1990: 211 [Sierra Leone]
				*Pseudomordellistena*	Ermisch, 1958: 386
			*Mordelloides*		Ray, 1939 Ray, 1941: 277
			*Mordellopalpus*		Franciscolo, 1955: 179
			*Neocurtimorda*		Franciscolo, 1949: 1 (Doriana, 1949: 1–4) Franciscolo, 1950 Franciscolo, 1955: 171 Franciscolo, 1990: 207 [Sierra Leone]
			*Neoglipa*		Franciscolo, 1952 Franciscolo, 1952a: 332 [New Guinea Island]
				*Macroglipa*	Franciscolo, 1952: 332 [New Guinea Island]
			*Stenoglipa*		Franciscolo, 1952: 344 [New Guinea Island]
			*Neotomoxia*		Ermisch, 1950: 10
			*Ophthalmoglipa*		Franciscolo, 1952: 353 [New Guinea] (Doriana, 1952: 2) Franciscolo, 1955: 1052 Franciscolo, 1959: 23
				*Stenoglipa*	Takakuwa & Masatoshi, 2000: 64
			*Paramordella*		Píc, 1936: 259
			*Paramordellana*		Ermisch, 1968: 265 Ermisch, 1950: 59
			*Paramordellaria*		Ermisch, 1968 Lu & Jackman & Johnson, 1997: 746
			*Paraphungia*		Ermisch, 1969: 4
			*Parastenomordella*		Ermisch, 1950: 60 [Brazil: Brasilien]
			*Paratomoxia*		Ermisch, 1950: 5 Tokeji, 1954: 3
				*Metatomoxia*	Chujo, 1957: 1
			*Paratomoxioda*		Ermisch, 1954
			*Phungia*		Píc, 1922: 15, 17 Ermisch, 1950: 49
			*Plesitomoxia*		Ermisch, 1954: 170 Ermisch, 1955 Franciscolo, 1990: 207 [Sierra Leone]
			*Praemordella*		Shchegoleva-Barovskaya, 1929: 27
			*Pseudomordellaria*		Shchegoleva-Barovskaya, 1929: 27
			*Pseudotomoxia*		Ermisch, 1950: 52
			*Sphaeromorda*		Franciscolo, 1950
			*Stenaliamorda*		Ermisch & Chûjô, 1968
			*Stenomorda*		Ermisch, 1950: 17
			*Stenomordella*		Ermisch, 1941: 115 Ermisch, 1941: 115–117 Ermisch, 1950: 57
			*Stenomordellaria*		Ermisch, 1950: 57 Ermisch, 1950: 34–92 Franciscolo, 1981: 205
			*Stenomordellariodes*		Ermisch, 1954: 92
			*Succimorda*		Kubisz & Daniel, 2001: 273
			*Tolidomordella*		Ermisch, 1950: 69 Lu, Wenhua & Jackman & Johnson, 1997: 752
			*Tolidomoxia*		Ermisch, 1950: 13
			*Tomoxia*		Costa, 1854: 8 Smith, 1882: 78 [America] Costa, 1884: 8 Champion, 1898: 84 Nicolay, 1914: 30 Chujo, 1935: 78 [China: Taiwan; Japan; Philippines; Papua, Australia, Europe, Mexico, Southern America] Liljeblad, 1945: 54 [North America, Europe] Ray, 1949: 285 [Fiji] Ermisch, 1950: 52 Buck, 1954: 16 Ermisch, 1956: 298 Franciscolo, 1956: 2 Downie, 1996: 1156 [North-Eastern America, North-Eastern Mexico] Lu & Jackman & Johnson, 1997: 743 Takakuwa, 1999: 1
				*Metatomoxia*	Chujo, 1957: 1
			*Tomoxioda*		Ermisch, 1950: 53
			*Trichotomoxia*		Franciscolo, 1950: 130 Franciscolo, 1955: 1052 Franciscolo, 1955: 164
			*Variimorda*		Méquignon, 1946 Ermisch, 1956:298 Horak, 1985: 4 Serrahima, 2007: 560
				*Galeimorda*	Horak, 1985: 15
				*Sulcatimorda*	Batten, 1977: 167
			*Variimorda*		Mequignon, 1946 Serrahima, 2007: 560
			*Wittmerimorda*		Franciscolo, 1952: 69
			*Yakuhananomia*		Kônô, 1935: 124 [Japan] Fauna, 1936: 25 [Japan] Ermisch, 1950: 55 Lu, 1997: 747
			*Zeamordella*		Broun, 1886: 84 Ermisch, 1950: 70 Franciscolo, 1981: 191 Franciscolo, 1992: 569
		Mordellistenini			Ermisch, 1941 Franciscolo, 1955: 1062 Franciscolo, 1956: 3 Franciscolo, 1956: 225 Franciscolo, 1959: 23 Franciscolo, 1962: 101 Nomura, 1967: 15 Nomura, 1967: 15 Serrahima, 2007: 560 Horak, 2009: 209
			*Asiatolida*		Shiyake, 2000: 26
			*Calyce*		Champion, 1891: 307 Champion, 1898: 92 Blair, 1922: 221 Ray, 1941: 284 Ermisch, 1943: 53–60 Ermisch, 1950: 87
			*Calycemorda*		Ermisch, 1969: 304
			*Calyceoidea*		Ermisch, 1969: 307
			*Dellamora*		Normand, 1916: 284 Ermisch, 1941:711, 721, 722 Van Zwaluwenburg, 1948 [Solomon Islands] Ray, 1949: 304 Ermisch, 1950: 80
			*Diversimorda*		Ermisch, 1969: 301
			*Ermischiella*		Ermisch, 1941: 724 Liljeblad, 1950 Franciscolo, 1950: 2 Franciscolo, 1950
			*Fahraeusiella*		Ermisch, 1953: 316
			*Falsomordellina*		Nomura, 1966: 44
			*Falsomordellistena*		Ermisch, 1941: 715, 725 Ermisch, 1950: 83 Tokeji, 1954: 14
				*Falsomordellistena*	Lu, 2009: 119
				*Falsomordellistenoda*	Ermisch, 1950a: 85
				*Palaeostena*	Kubisz, 2003: 185 (Eocene Fossil)
			*Falsopseudomoxia*		Franciscolo, 1965
			*Glipostena*		Ermisch, 1941: 715, 723 Ermisch, 1950: 80 Tokeji, 1954: 19 Franciscolo, 1962: 118 Franciscolo, 1999: 101 Franciscolo, 1999: 242
			*Glipostenoda*		Ermisch, 1950: 81 Ermisch, 1949–1950: 45, 81 Krefeld, 1949–50, 45–46: 80 Franciscolo, 1958: 71 Franciscolo, 1962: 121 Lu, 1997: 761 Lu, 2009: 121
			*Gymnostena*		Franciscolo, 1950: 7
			*Mordellina*		Schilsky, 1908: 137 Franciscolo, 1962: 107 Ermisch, 1965: 199 Batten, 1990: 150 [Papua New Guinea] Franciscolo, 1990: 211 [Sierra Leone]
				*Mordellina*	Ermisch, 1967: 133
				*Pseudomordellina*	Nomura, 1966: 49
				*Pseudomordellistena*	Ermisch, 1952a: 145 Ermisch, 1965: 199 Batten, 1990: 150 [Papua New Guinea] Franciscolo, 1990: 211 [Sierra Leone]
			*Mordellistena*		Linne, 1758: 420 Costa, 1853 Costa, 1854: 16, 31 Helmuth, 1864: 105 [America: Illinois] Broun, 1880: 415 Costa., 1882: 85 [America: New York] Smith, 1883: 80, 81 [America] Champion, 1895: 271 [North- Western Australia] Champion, 1896: 50 Kolbe, 1897: 254 Champion, 1898: 92 Webster, 1901: 176 Dury Charles, 1906 [North America] Nicolay, 1914: 30 Champion, 1917: 181 Kono, 1928: 37 [Japan] Stshegoleva-Barovskaja, 1930: 57 Scegoleva-Barovskaja, 1931: 419 Ray, 1941: 286 [Mexico, Guatemala] Ermisch, 1941: 711, 716 Liljeblad, 1945: 68 Breuning, 1948: 503–523 Ray, 1949: 286 [Fiji] Ermisch, 1950: 76 Ermisch, 1950: 52 Ermisch, 1950: 31 Pic, 1950: 148 Pic, 1952: 106 Buck, 1954: 16 Tokeji, 1954: 5
					Franciscolo, 1955: 1062 Ermisch, 1956: 305 Franciscolo, 1956: 3 Franciscolo, 1956: 232 Franciscolo, 1959: 23 Franciscolo, 1962: 101 Compte, 1969: 110 Horak, 1980: 285 Odnosum, 1989: 333 Franciscolo, 1990: 211 [Sierra Leone] Downie, 1996: 1159 [Northern America, Northern Mexico] Lu, 1997: 756 Serrahima, 2007: 560
				*Mordella*	Blair, 1921: 281
				*Mordellikoiles*	Franciscolo, 1942:133
				*Mordellina*	Schilsky, 1908: 137 Ermisch, 1950: 50
				*Mordellistena*	Costa, 1854 Emery, 1876: 81 Chujo, 1935: 80 Franciscolo, 1955: 1062 Ermisch, 1956: 305 Franciscolo, 1956: 232 Ermisch, 1963: 55 Serrahima, 2007: 560 Lu, 2009: 122
				*Mordellistenula*	Stshegoleva-Barovskaja, 1930: 56
				*Mordellochroa*	Emery, 1876: 80 Champion, 1898: 92
				*Pseudomordellina*	Ermisch, 1952: 47 Franciscolo, 1955: 1076 Ermisch, 1956: 311 Franciscolo, 1956: 240 Franciscolo, 1959: 27
				*Pseudomordellistena*	Ermisch, 1951(1952): 143, 145 Nakane, 1957: 54 Nomura & Kato, 1957: 81
			*Mordellistenalia*		Ermisch, 1958: 376 [Congo]
			*Mordellistenochroa*		Horák, 1982
			*Mordellistenoda*		Ermisch, 1941: 589 Ermisch, 1941: 715, 722 Ermisch, 1950: 87
			*Mordellistenula*		Stchegoleva-Barowskaja, 1930 Stshcgolewa-Barowskaja, 1931: 57 Ermisch, 1941: 711, 718, 719 Ermisch, 1950: 76 Ermisch, 1956: 305 Compte, 1969: 114
			*Mordellochroa*		Emery, 1876: 80 Ermisch, 1941: 714, 717 Franciscolo, 1950: 6 Ermisch, 1950: 78–79 Tokeji, 1954: 12 Ermisch, 1956: 312 Kazab, 1979: 83 Odnosum, 1996: 47
			*Mordellochroidea*		Ermisch, 1969: 311
			*Mordelloxena*		Franciscolo, 1950b: 6
			*Morphomordellochroa*		Emery, 1876 Ermisch, 1969: 308 Serrahima, 2007: 560
			*Neomordellistena*		Ermisch, 1950: 52
				*Neomordellistena*	Batten, 1981: 348
			*Palmorda*		Ermisch, 1969: 312
			*Palpomorda*		Ermisch, 1969
			*Paramordellistena*		Ermisch, 1950: 73
			*Phunginus*		Píc, 1922
			*Pselaphokentron*		Franciscolo, 1955: 184
			*Pseudodellamora*		Ermisch, 1941: 714, 720 Ermisch, 1942 Ermisch, 1950: 80
			*Pseudotolida*		Ermisch, 1950: 86
			*Raymordella*		Franciscolo, 1956: 227
			*Rolcikomorda*		Horak, 2008: 67–68 [Madagascar]
				*Hauckina*	Horak, 2008: 72 [Madagascar]
			*Tolida*		Mulsant, 1856 Ermisch, 1941 Franciscolo, 1942: 8 Ermisch, 1956: 312
			*Tolidopalpus*		Ermisch, 1951 Ermisch, 1952: 144, 149 Shiyake & Shigehiko, 1995: 12
			*Tolidostena*		Ermisch, 1942: 674 Ermisch, 1950: 79 Kiyoyama & Yoshimi, 1991: 53
				*Neotolidostena*	Kiyoyama & Yoshimi, 1991: 53
				*Tolidostena*	Kiyoyama & Yoshimi 1991: 54
			*Uhligia*		Horák, 1990: 141
			*Xanthomorda*		Ermisch, 1968 Ermisch, 1969 Batten, 1990: 143 [Papua New Guinea]
		Reynoldsiellini			Franciscolo, 1957: 237
			*Reynoldsiella*		Ray, 1930: 164 Ermisch, 1950: 48
		Stenaliini			Franciscolo, 1955: 1056 Franciscolo, 1956: 219
			*Brodskyella*		Horák, 1989: 37
			*Pselaphostena*		Franciscolo, 1950: 130 Franciscolo, 1952: 456
			*Stenalia*		Mulsant, 1856: 83, 387 Champion, 1898: 92 Ermisch, 1950: 76 Franciscolo, 1955: 1056 Ermisch, 1956: 305 Franciscolo, 1956: 220 Horak, 1978: 402 Horak, 1995: 89
			*Stenaliodes*		Franciscolo, 1956: 219
